# Transformation toward precision large-scale operations for sustainable farming: A review based on China’s pig industry

**DOI:** 10.5455/javar.2024.k859

**Published:** 2024-12-29

**Authors:** Md Kamrul Hasan, Hong-Seok Mun, Keiven Mark B. Ampode, Eddiemar B. Lagua, Hae-Rang Park, Young- Hwa Kim, Md Sharifuzzaman, Chul-Ju Yang

**Affiliations:** 1 Animal Nutrition and Feed Science Laboratory, Department of Animal Science and Technology, Sunchon National University, Suncheon, South Korea; 2 Department of Poultry Science, Sylhet Agricultural University, Sylhet, Bangladesh; 3 Department of Multimedia Engineering, Sunchon National University, Suncheon, South Korea; 4 Department of Animal Science, College of Agriculture, Sultan Kudarat State University, Tacurong, Philippines; 5 Interdisciplinary Program in IT-Bio Convergence System (BK21 Plus), Sunchon National University, Suncheon, South Korea; 6 Interdisciplinary Program in IT-Bio Convergence System (BK21 Plus), Chonnam National University, Gwangju, South Korea; 7 Department of Animal Science and Veterinary Medicine, Bangabandhu Sheikh Mujibur Rahman Science and Technology University, Gopalganj, Bangladesh; † Both authors have equal contributions to this work as co-first authors.

**Keywords:** China, Pig farm operation, Smart agriculture, Sustainability

## Abstract

This review evaluates the current situation of pig farming, identifies challenges, and projects for the sustainable development of the Chinese pig industry. A literature review using keyword searches was conducted on Google Scholar for articles from 2017–2023. The review included studies focused on pig farming in China, covering prospects, challenges, quantitative data on production, marketing, and consumption, automation in livestock farming, and publications from peer-reviewed journals, credible websites, government reports, and conference proceedings. Pork consumption in China is increasing, and the country imports a sizable amount of pork annually. Even though small-scale farms still account for most operations, the pig industry is undergoing a critical stage of modernization and transition towards large-scale farming. The major challenges identified were feed, disease, antimicrobial resistance, environmental pollution, and pork prices. Smart technologies, such as cameras, Internet of Things, and sensors, integrated into precision pig farming can improve productivity and animal health through real-time data collection and decision-making. To solve the problems we face now, we need to put a lot of money into large-scale transformation, the creation of new animal precision tools, the automation of manure treatment, and the research and development of long-lasting alternative energy sources like photovoltaics and wind. By implementing these strategies, large-scale precision pig farming in China can become economically and environmentally sustainable, which can ultimately benefit consumers by supplying wholesome pork products.

## Introduction

In 2023, pork became the most consumed meat globally, making up 42% of total meat consumption, outpacing beef (37%) and chicken (21%) [1]. China, the USA, and the European Union (EU) emerged as the top three pork-producing regions, with China leading the pack. In 2022, China produced 55.41 million tons of pork annually, followed by the USA at 22.46 million tons and the EU at 12.25 million tons [2,3]. Despite its massive production, China remains the largest importer of pork due to its high domestic demand [2]. For instance, in 2023, China’s pork production reached 55.5 million tons, yet consumption surpassed production at 57.58 million tons [3]. This imbalance is projected to persist, with production forecasted to reach 55.95 million tons and consumption climbing to 57.73 million tons by 2024 [3]. Looking ahead to 2029, China’s pork production and consumption are expected to rise to 59.72 and 60.77 million tons, respectively [4].

Pork is a vital source of animal protein in China, forming the backbone of the country’s meat consumption [5]. Per capita pork consumption saw a sharp rise from 18.2 kg in 2020 to 25.2 kg in 2021, representing 76.60% of total meat consumption that year [6]. By 2029, this figure is projected to increase further to 42.3 kg per person [4]. Despite this growing demand, the vast majority of pig farms in China (99.12%) continue to operate on a small, conventional scale [7]. These traditional farming methods present significant environmental challenges, including degradation that affects both rural and urban areas [8].

In response to growing domestic demand and rapid urbanization [9], China’s pig farming industry is transitioning from small-scale, traditional farms to large-scale, industrialized systems [10]. This shift offers significant benefits, including increased efficiency, higher production capacity, and reduced production costs [11,12]. To fully capitalize on these advantages, precision farming technologies are being integrated into large-scale operations. Precision farming tools (PLFs), such as cameras, sensors, and Internet of Things (IoTs) devices, enable real-time livestock monitoring, early disease detection, and automation of key processes like feeding and waste management [13–17]. This not only boosts productivity and resource efficiency but also enhances environmental sustainability by minimizing resource waste and reducing the ecological impact of large-scale farming. By linking large-scale farming with precision technology, China can meet rising pork demand while ensuring sustainable and efficient production practices.

This review seeks to examine the key factors and challenges shaping the future of pig farming in China, with a special focus on the potential of precision farming. While many obstacles are well-documented, discussions on how precision technologies can address these issues remain limited. The first section of this review covers the recent expansion of pig farming in China. The second and third sections discuss the challenges within the industry and recent global advancements in precision pig farming. The final sections offer insights into innovations and prospects for large-scale, sustainable pork production.

## Materials and Methods

### Searching and selecting articles

A literature review was conducted to gather relevant articles. The review followed a structured process that involved the definition of exclusion and inclusion criteria, the selection of a database, and the application of keywords for a targeted search. The database used for the literature search included Google Scholar, covering a period from 2017 to 2023 to ensure recent developments were captured. Non-academic sources such as government reports, international project documents, and policy papers were also included to offer a more comprehensive view.

### Keyword search

The search employed a combination of the following keywords: “large-scale,” “pig farming,” “China,” “sustainability,” “challenges,” “opportunity,” and “precision.” The Boolean operator “AND” was used to refine and expand the searches, ensuring relevant literature was identified.

### Inclusion criteria

To focus on the relevant literature, the following inclusion criteria were applied:

Studies focused on the prospects and challenges of pig farming in China, including government policies and technological advancements.

Quantitative data on pig production, marketing, and consumption.

Articles discussing mechanization and precision farming in livestock production, especially in the pig sector.

Peer-reviewed academic journals, official government reports, and reliable non-academic sources in both Chinese and English were included. Information written in Chinese was translated into English using Google Translate.

### Exclusion criteria

To ensure quality and relevance, the following exclusion criteria were applied:

Articles published before 2017 or after 2023 were excluded to focus on the most recent technological and policy changes.

Non-peer-reviewed articles or those lacking scientific rigor (such as those with small sample sizes or weak statistical methods) were excluded.

Studies focusing on traditional or small-scale farming methods were filtered out unless they offered significant comparative insights relevant to large-scale farming.

Articles on non-pig farming systems, such as poultry, cattle, or crop farming, were not included in the review.

Publications are not available in English or Chinese were excluded, as well as studies that lacked sufficient credibility or depth of analysis.

### Article filtration process

The article filtration process involved several steps, from initial identification to the final selection, ensuring that only the most relevant and high-quality studies were included. The process was designed to systematically narrow down the search results based on predefined inclusion and exclusion criteria, using a combination of academic rigor, relevance to large-scale pig farming, and a specific focus on precision farming in China. An initial pool of 280 articles was identified through database searches. After applying the inclusion and exclusion criteria and conducting a title and abstract screening, 159 articles were shortlisted for full-text review. In full-text review, each study was carefully evaluated based on its depth of analysis, methodological rigor, and relevance to the study’s objectives. A detailed assessment was carried out to ensure the selected articles provided credible and peer-reviewed data. After the full-text review, 27 studies were deemed to meet the selection criteria and were included in the final analysis. Articles were further scrutinized based on the quality of data, methodology, and relevance to large-scale precision pig farming.

## Current Status of Pig Farming in China

### Pig population distribution

**Figure 1.**
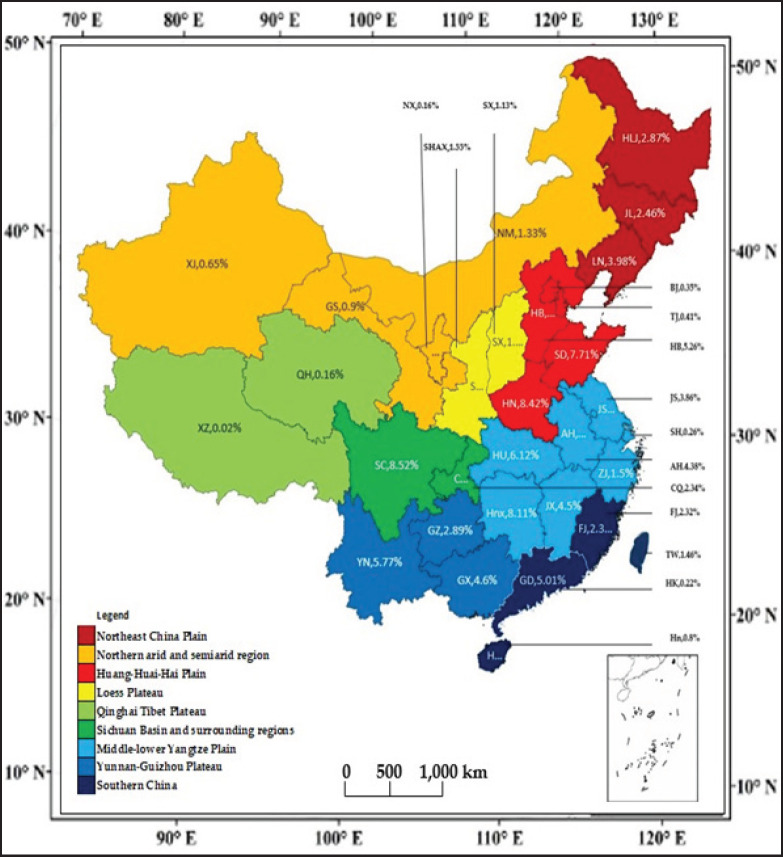
Pork production rates across China’s agricultural regions and provinces in 2017. Generated from [10,19,20]. The provinces’ abbreviations are provided in Table 1.

The pig industry shifted towards increased production [10], although pork output dipped in 2007 due to porcine reproductive and respiratory syndrome (PRRS) and rising grain prices [18]. Variations in climate and agricultural systems across China affect pig populations, with the Middle-Lower Yangtze Plain leading production in 2017 (28.73%), followed by the Huang-Huai-Hai Plain (22.14%) and Yunnan-Guizhou Plateau (13.26%) (Fig. 1) [10,19,20]. The lowest production was in the Qinghai-Tibet Plateau (0.18%) (Fig. 1).

**Figure 2.**
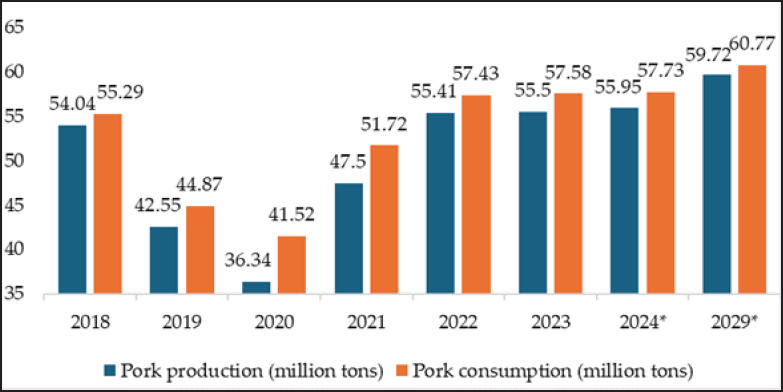
Pork production and consumption in China from 2018 to 2023 [3]; * predicted value for 2024 [3] and 2029 [4].

**Figure 3.**
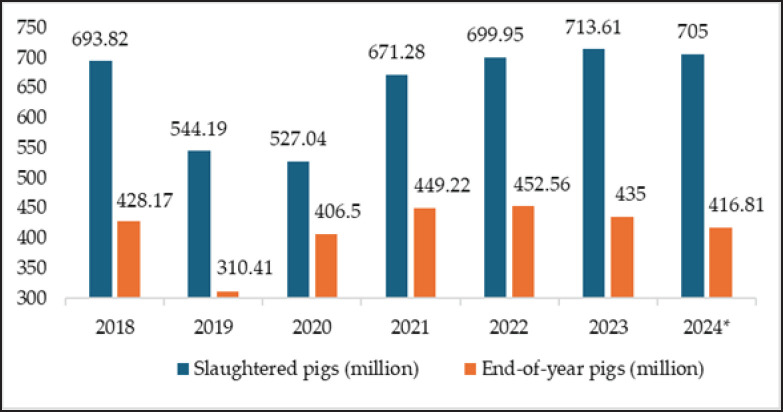
Number of slaughtered and end-of-year pigs in China between 2018 and 2023 [21]. * predicted value for 2024 [21].

China’s pork production drops from 54.04 million tons in 2018 to 36.34 million tons in 2020, then rises to a projected 55.95 million tons by 2024 (Fig. 2). Consumption follows a similar trend, reaching 57.73 million tons in 2024 (Fig. 2). Between 2018 and 2024, China’s pork production, along with metrics such as consumption, number of slaughtered pigs, and end-of-year pigs’ figures (including predicted values), fluctuated (Figs. 2 and 3) [21] due to factors such as African swine fever (ASF) [22], which caused a notable decrease in production and slaughtered pigs in 2019 (Figs. 2 and 3), raising pork prices [23]. In response, the government implemented policies to stabilize and increase pork production from 2021 onward [24].

**Table 1.** Provinces with their abbreviations and regions.

Province name	Abbreviations	Region
Heilongjiang	HLJ	
Jilin	JL	Northeast China Plain
Liaoning	LN	
Xinjiang Uygur	XJ	
Gansu	GS	Northern arid and semiarid region
Nei Mongol	NM	
Ningxia Hui	NX	
Beijing	BJ	
Tianjin	TJ	
Hebei	HB	Huang-Huai-Hai Plain
Shandong	SD	
Henan	HN	
Shanxi	SX	Loess Plateau
Shaanxi	SHAX	
Xizang	XZ	Qinghai Tibet Plateau
Qinghai	QH	
Chongqing	CQ	Sichuan Basin and surrounding regions
Sichuan	SC	
Shanghai	SH	
Jiangsu	JS	
Zhejiang	ZJ	
Anhui	AH	Middle-lower Yangtze Plain
Jiangxi	JX	
Hubei	HU	
Hunan	Hnx	
Guizhou	GZ	
Yunnan	YN	Yunnan-Guizhou Plateau
Guangxi	GX	
Fujian	FJ	
Guangdong	GD	
Hainan	Hn	Southern China
Taiwan	TW	
Hong Kong	HK	

**Figure 4.**
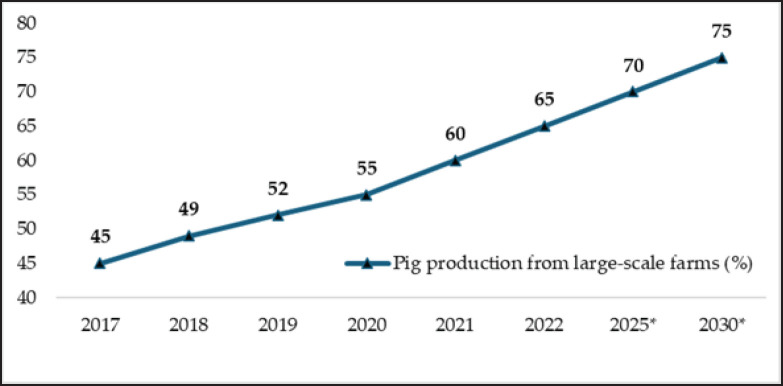
Percentage of China’s annual pig production (2017-2022) from large-scale farms (produce >500 pigs a year) [7]; * predicted values for the years 2025 and 2030 [7].

Most pig farms (99.12%) in China are small-scale [7], with local breeds such as Laiwu and Jinhua commonly raised for their meat quality [25]. However, large-scale farms, which use commercial breeds like Duroc and Landrace, dominate pork production, contributing 90%–95%, while local breeds contribute only 5%–10% [26]. Large farms are projected to account for 70% of total pig production by 2025 (Fig. 4).

### Large-scale pig production system

Large-scale pig farms in China are divided into two models: the enterprise-farmer model and the self-support model [27,28]. The enterprise-farmer model is most common, involving companies providing inputs like piglets and feed, while farmers rear the pigs. The self-support model, though more capital-intensive, allows for full control over production [27,28]. Leading companies like Wens Foodstuff Group, Muyuan Foods, Zhengbang Group, New Hope Group, and Charoen Pokphand Group use vertical integration and contract farming, contributing significantly to the market [29]. In 2019, these firms produced 41.7 million pigs, representing 7.69% of the total market share [28].

### Pig farming insurance and agricultural cooperatives

Farmers manage risks through agricultural insurance and cooperatives [30]. Membership in cooperatives positively influences high-quality pork production, especially for smaller farms with less experience [30]. Cooperatives also encourage the purchase of insurance to mitigate production risks, and participation is often influenced by farmers’ education, experience, and government trust [30].

### Alteration in pig production systems and its effects on rural livelihoods

The shift toward large-scale farming has reduced small farms and rural households engaged in pig rearing [31]. Between 2007 and 2017, large and medium farms increased by 145% and 60%, respectively, while small farms declined by 54% [10]. This transition reshaped rural livelihoods, and pig-rearing households dropped by 74.65% from 1996 to 2016 [31].

### Implementation of smart technologies

China is advancing smart farming technologies such as IoT, automation, and artificial intelligence (AI) to modernize pig farming [32]. Innovations include precision feeding [33,34], early disease diagnosis [35], and automated environmental control [14,36]. IoT technologies, industrial internet, and next-generation AI support better management, monitoring, and decision-making in pig farms, improving productivity and efficiency across large-scale operations [37–40].

## Challenges of Pig Farming

### Feed

The pig farming industry faces feed shortages, particularly in corn and soybeans. Despite increased domestic production between 1961 and 2020 [12], demand still outstrips supply, necessitating significant imports [41,42]. In 2020, China imported 100.33 million tons of soybeans [41,42] and 18.71 million tons of corn in 2022 [43]. Feed prices have risen due to reliance on imports, which account for over 70% of pig production costs [44,45]. Swill feeding (feeding pigs food waste or kitchen scraps), once used in small-scale farms to cut costs, has been banned since 2018 due to ASF concerns [46]. Large-scale pig and soybean farming could provide a sustainable solution to balancing feed and meat security [12], and the government has introduced policies to boost domestic production [47].

### Land

With an average of 0.38 hectares of cultivated land per household [48], the expansion of large-scale pig farming demands more space. A 26-floor pig farm tower in Hubei province, which houses units for all stages of pig production, exemplifies vertical farming as a potential land-efficient solution [49].

### Epidemic threats

Pigs are vulnerable to diseases such as PRRS, ASF, foot and mouth disease, classical swine fever, and porcine epidemic diarrhea [50,51]. *Actinobacillus pleuropneumonia*, *Salmonella*, and *Escherichia coli* are the main bacteria that pose a threat to pig production [52]. In 2018, ASF caused the death or culling of 350,000 pigs, leading to a 72.49% decrease in pig numbers [51]. Despite vaccine development, challenges remain due to issues like inadequate potency [53,54] and difficulties with multi-age pig rearing [53,55]. Inadequate diagnostic facilities and insufficient training among farm staff exacerbate the spread of disease [56], especially in small-scale operations [18].

### High proportion of small-scale farms

**Figure 5.**
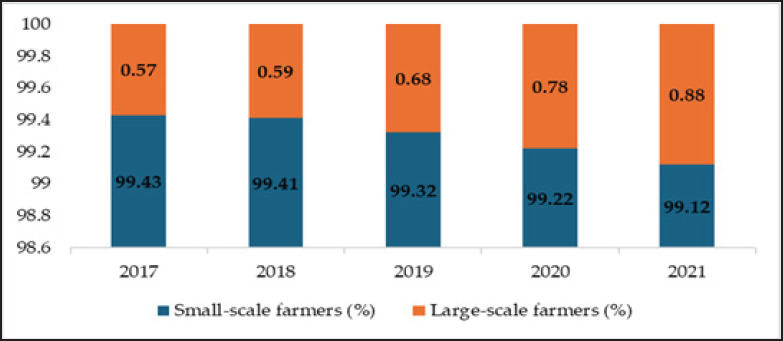
Variations in China’s percentage of small-scale farmers (those who raise 1–500 pigs a year) and large-scale farmers (those who raise >500 pigs a year) between 2017 and 2021 [7].

Small-scale farms dominate China’s pig farming industry, though their numbers are declining (Fig. 5). However, they still accounted for 99.12% of all pig farms in 2021 (Fig. 5). Small-scale farms face challenges in adopting precision technologies [57], resulting in lower economic benefits compared to large-scale operations [58]. Moving forward, more small-scale farmers are expected to collaborate with enterprises to improve production efficiency.

### Labor

Rural labor shortages due to urban migration have hit pig farming hard [59], with 81% of large-scale farms facing shortages during COVID-19 [60]. Rising labor costs, from 197 RMB ($28.84) in 2009 to 498 RMB ($80.84) in 2014 [31], are pushing smaller farmers out of the industry. In 2017, labor costs were 4.1 RMB ($0.61) per kg of pig production for free-range farms, compared to just 1.5 RMB ($0.22) for large-scale farms [28], highlighting the efficiency of large-scale operations.

### Environmental pollution

China, one of the most populous countries, has high GHG emissions and severe water scarcity [61,62]. Small-scale farms produce higher CO_2_ emissions per kg of pork than large-scale operations [63,64]. Improper waste management exacerbates pollution [64], and the government’s efforts to recycle pig waste into farmland [65] have been largely ineffective due to farmers’ preference for chemical fertilizers [64].

### Pork price

Pork is a staple in China and significantly impacts the consumer price index [66]. Pork prices saw considerable fluctuations from 2017 to 2022 [67]. Initially stable at 20–30 RMB/kg ($3.01–4.51/kg) from 2017 to 2018, prices soared to 50–55 RMB/kg ($7.52–8.27/kg) in 2019 due to the ASF outbreak. This upward trend peaked at 60 RMB/kg ($9.02/kg) in 2020, driven by ongoing supply shortages. Afterward, prices declined to an average of 30–40 RMB/kg ($4.51–6.02/kg) in 2021 and approximately 28 RMB/kg ($4.21/kg) in 2022. The primary factors behind these fluctuations were the ASF outbreak and the resulting supply constraints [67].

### Antimicrobial resistance

China is the world’s largest consumer of veterinary antibiotics [68], leading to rising antibiotic resistance in animals [69]. Overuse of antibiotics, particularly in small-scale farms where disease detection is delayed, poses a serious risk [70]. Efforts to reduce antibiotic use, including the ban on adding antimicrobials to feed in 2020 [71], aim to combat this growing problem.

### Strict environmental protection policy

The government has enacted strict policies to reduce pollution from livestock farming [72–75]. However, these policies have led to the closure of many pig farms, especially those near water sources, and have modestly reduced environmental damage at a high financial cost [76]. Regulations have decreased pig production and raised domestic pork prices [76].

### Global Advancement in Precision Farming

The demands of contemporary large-scale livestock farming may not be met by conventional management techniques, which depend only on farmers’ observations, discretion, and experience. PLF uses the real-time analysis of tracking data, images, sounds, weight, body condition, and biological metrics in livestock to remotely identify and monitor the health and welfare of individual animals [77,78]. The expansion of large-scale pig production necessitates the development of automated and farmer-friendly animal identification systems that link animal data to precision management systems [79]. RFID [80,81], optical character recognition [82], and facial recognition [83,84] are used for individual pig identification and monitoring. Digital transformation not only provides personalized care and monitoring but is also promising for meeting the increasing food demand in the future [85]. Innovation and digital technologies aimed at improving productivity have been developed [86]. ICT tools are used for animal tracking using accelerometers [87] and global positioning systems [88], calculating feeding time using RFID [81], measuring daily water intake using flow meters [89], and detecting stress [90] and respiratory problems using sound analyzers [91,92]. These devices assist in real-time data collection that can be automatically analyzed using AI or statistical software [79]. Body temperature and animal identification can be measured using camera systems with built-in sensors [93,94]. In addition, cameras are useful for determining the body weight, lameness, and injuries [95–97]. Infrared cameras and 24/7 monitoring cameras have been used to detect estrus [98,99] and assess fertility [100,101]. Deep learning models are used to identify animals [102], posture [103,104], abnormal behavior or disease symptoms [105], feeding behavior [105], body weight [106,107], and water intake [108]. Modern pig farmers may need to use sensors and/or devices for precision pig farming to monitor the behavior and health of pigs and the general farm environment [109,110]. Robotic systems have proven profitable in that they save money, labor, and time when used for tasks such as cleaning and washing farms and carcass processing [111]. Decision-making and more accurate predictions can be facilitated by integrating sensor-driven data, AI, digital twins, and other cutting-edge technologies [14,111,112]. Using predictive analytics, farmers can reduce risks, foresee problems, and make well-informed decisions that will increase pig production and profitability. Knowing when their pigs are ready for shipping to a butcher shop can help producers develop a more accurate plan [113]. Although numerous smart technologies exist, only a few have been fully validated [114].

PLF incorporates health status monitoring and disease control measures [35,115–118]. Automatic monitoring of health parameters is the most effective and practical way to maintain the well-being of large herds of pigs [117]. Alterations in an animal’s drinking or eating behavior can indicate its overall health status because physiological disorders, unfavorable environmental circumstances, and diseases disrupt the animal’s normal drinking and/or feeding behavior [119,120]. RFID systems [121–123] and deep learning techniques [124–126] can be used to monitor drinking or eating behavior, including drinking or eating frequency. Recently, microfluidics has gained popularity owing to its rapid disease detection capabilities [127–129] and has proven to be a reliable and effective way to diagnose metabolic diseases (ketosis) [130]. Respiratory disorders, stress, and other illnesses can be recognized by animal sounds [131–133]. For respiratory diseases such as pneumonia, coughing sounds may be helpful indicators [134]. Yin et al. [92] developed an AlexNet model for cough recognition with 96.8% accuracy. In terms of real-time pig disease diagnosis, Exadaktylos [135] observed 85% using sound analysis. Sound can serve as an indicator of indoor air pollution on farms, providing a novel method for assessing environmental quality [136]. However, the primary limitation of using audio-based health assessments for livestock is the noisy environment prevalent on farms [111]. Sensors have been used successfully to identify foodborne illnesses in animals [137,138]. ZigBee-based networks can be used for the earlier detection of *Salmonella enteritidis* and *Escherichia coli* infections in piglets [139]. In addition to providing financial benefits, early disease detection and treatment also improve animal welfare, which is essential for sustainability [140] and entering foreign markets [141]. Early disease detection techniques must be used in China because disease incidence and recurrence are common in large-scale pig farms. China can potentially enhance precision pig farming by using highly accurate and validated sensors and/or devices; however, the domestic production of sensors and/or devices is necessary to support long-term maintenance and decrease reliance on foreign sources.

While PLF offers many advantages, several significant drawbacks must be addressed to maximize its potential. High upfront costs [85] can discourage farmers from adopting these technologies, as the technical complexity [142] demands specialized training and precise, expert installation of animal-attached sensors [133] to function effectively. Moreover, there are challenges in managing the vast amounts of data generated [143,144], alongside concerns about data ownership [142], privacy, and security. Additionally, over-reliance on technology may cause farmers to lose their practical skills in farm operations [145], and the continuous internet access required for PLF tools poses challenges, especially in rural areas [146]. Furthermore, research is limited, and on-farm applications are still in the embryonic stage [147]. There are also significant animal welfare concerns, especially for human-animal relationships [148], as these tools often prioritize data collection without adequately addressing the emotional well-being of the animals. Therefore, to fully harness the capabilities of PLF tools, it is crucial to effectively tackle these challenges.

### Prospect of Precision Large-Scale Pig Farming

The MARA (Ministry of Agriculture and Rural Affairs) published the China Agricultural Outlook (2020–2029) [4], forecasting a 1.9% annual increase in pork production. By 2035, the demand for live pigs is projected to nearly match supply, with slaughter estimates reaching around 374 million [149]. This positive shift is underpinned by the industry’s transition from traditional to large-scale digital operations [149]. This transformation has not only boosted farmers’ incomes [12] but also enhanced worker productivity and the growth rates of pigs [58].

To address environmental concerns, a shift from low-intensive to high-intensive farming practices is essential [150–152]. Projections indicate that large-scale farms will make up 75% of pig production by 2030 (Fig. 4). Concurrently, digital technology adoption in animal husbandry is expected to reach 75% by 2035 [153]. Innovations like precision feeding are improving nutrient efficiency, leading to a reduction in GHG by 6% [154], protein intake by 25%, and N_2_ and P excretion by 40% [155,156]. These advancements can also lower feed costs by over 8% [157].

As the pig industry evolves, the need for environmental monitoring intensifies. By 2035, the sector will require about 70.12 million environmental sensors [149]. The breeding sector alone is projected to need 129 million by 2035, potentially creating a market valued at around 86 billion RMB ($12.04 billion) [149]. The integration of AI and digital technologies has significantly improved the monitoring of pig health and welfare in China. For instance, a convolutional neural network-based model used on pig farms for sow estrus sound monitoring achieved an impressive 97.52% accuracy [158]. Another study used machine vision systems to monitor individual feeding and drinking behaviors, where MobileNetV2 achieved a recall rate of over 97% [159]. Additionally, Ositanwosu et al. [160] used multilayer perceptron neural networks and 3D camera images to predict pig weight accurately based on abdominal circumference and age. Pan et al. [161] achieved a 98% accuracy rate in identifying diseased pigs using a combination of residual neural networks and Wasserstein Generative Adversarial Networks.

MARA’s initiatives, such as the “Technical Guidelines for Green Development of Agriculture (2018-2030)” [162] and the “Development Plan for Digital Agriculture and Rural Areas (2019–2025)” [163], aim to promote sustainable practices and incentivize technological adoption through financial support. The 2022 revision of the “Animal Husbandry Law of the People’s Republic of China” [164] mandates local governments to oversee livestock farms and enforce regulations against environmental pollution, further emphasizing the importance of precision farming.

Vertical integration within the pig supply chain is another strategy that can help reduce costs and stabilize supply by linking breeding with feed production [165]. This approach is anticipated to expand, supporting environmental conservation and minimizing price fluctuations. However, the extensive use of antibiotics in pig farming raises significant concerns about antimicrobial resistance [166]. MARA’s regulations since 2019, which ban growth-enhancing drugs except for specific herbal remedies, reflect a commitment to safer livestock management [71]. Moreover, by utilizing gene-editing technologies like zinc-finger nucleases, transcription activator-like effector nucleases, CRISPR, and base editors, coupled with advancements in animal genetics and biotechnology, researchers can develop new pig breeds that offer enhanced economic traits and disease resistance [167,168]. To overcome ethical concerns, a regulatory framework should be established that ensures product safety, animal welfare, and environmental sustainability [169].

Looking ahead, the establishment of more large-scale pig farms focusing on environmentally sustainable practices is expected [170]. Despite the ongoing promotion of sustainable manure management, approximately 51.5% of farmers have yet to adopt these techniques [171]. Stricter environmental regulations are likely to drive higher adoption rates. Notably, pig farming contributes 76.8% of wastewater and manure discharge [172], highlighting the urgent need for effective wastewater treatment technologies that comply with local standards.

Government support will be crucial for a successful transition to large-scale precision pig farming. This includes financial assistance to farmers, enhancements in infrastructure, organizing livestock technology fairs, assuring uninterrupted electricity and internet access, and training on PLF tools. Addressing the potential displacement of small-scale farmers due to the rise of larger operations is essential to maintaining skilled personnel in the industry and ensuring the sustainable development of China’s pig industry.

## Sustainable Large-Scale Pig Farming

Large-scale pig farming significantly reduces the marketing age of pigs, alongside lowering feed costs and the growth rate of feed consumption [12]. Between 2012 and 2020, the average marketing age for pigs across different farming scales was approximately 163 days for backyard farms, 155 days for small farms, 150 days for medium farms, and 143 days for large-scale operations [12]. Feed costs per pig during this period varied, with backyard farms at 2015 RMB ($309.52), small farms at 1875 RMB ($288.02), medium farms at 1800 RMB ($276.49), and large-scale farms at 1780 RMB ($273.43) [12]. Disease prevention costs also differed by scale, ranging from 17 RMB ($2.61) for backyard farms to 26 RMB ($3.99) for large farms [12]. The growth rate of feed consumption in 2018 was about 0.15 for backyard farms and decreased to 0.12 for large-scale farms [12]. In 2020, net profit per pig varied across scales, with backyard farms earning 1232.64 RMB ($178.64) and large-scale operations achieving 1619 RMB ($234.64) [12]. Between 2008 and 2020, the profit-to-cost ratio was higher in large-scale farming compared to backyard systems [12]. Wu et al. [173] assessed the environmental consequences from 1998 to 2020 and indicated that large-scale pig farming yields better economic benefits and generates less pollution compared to smaller operations. Implementing PLF tools in large-scale settings can facilitate early detection of health and welfare issues in pigs, potentially reducing disease prevention costs.

The Chinese government aims for net-zero carbon emissions by 2060 and peak emissions by 2030, highlighting the need to combat climate change [174]. Animal husbandry significantly contributes to GHG emissions due to outdated technologies and energy-intensive operations [175]. Electricity production accounts for 26.9% of GHG emissions, adversely affecting public health and the environment [176]. Technologies such as borehole thermal energy storage and photovoltaic-thermal systems can cut reliance on fossil fuels, potentially reducing CO_2_ emissions by 20,850 kg annually [177]. Innovative solutions like geothermal heat pumps and wireless intelligent infrared thermal control technology enhance energy efficiency in pig farms, reducing electricity consumption and lowering costs and GHG emissions [178,179].

**Figure 6.**
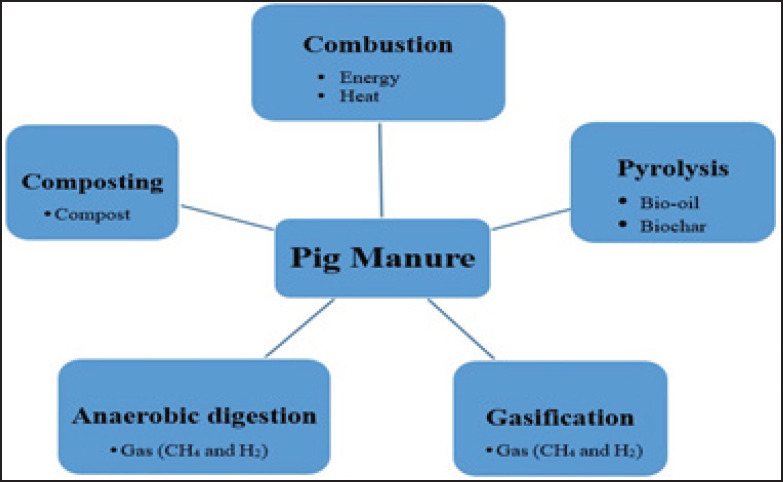
Technologies and processes for treating pig manure with its final products [195,196].

**Table 2.** Challenges and proposed methods/technologies for improving pig farming in China.

Challenges	Feed	Land	Strict environment protection policy	Pork price	Increase proportion of small-scale farms
Method/technology proposed	Precision feeding Technical support and subsidies	Expansion of vertical farming Precision pig farming technology	Precision pig farming technology Training of farmers	Vertical integration Use of renewable energy Precision pig farming technology	Financial and technical assistance Automation Training of farmers
Challenges	Labor	Environment pollution		Antibiotic resistance	Epidemic threats
Method/technology proposed	RFID, optical character and facial recognition, and camera- pig identification Camera- body weight measurement Accelerometers and GPS- pig tracking RFID- calculating feeding time Flow meters- measuring daily water intake Infrared and monitoring camera- estrus detection and assessing fertility Robotic systems- cleaning and washing farms	Borehole thermal energy storage GHP system Photo-voltaic and thermal power generation Precision feeding Slurry separation Combustion Pyrolysis Gasification Anaerobic digestion Composting Separation of liquid and solid fractions Integrated livestock-crop production		Use of antibiotic substitutes Guideline imply Disease-resistance pig breeds	Vaccination Sound analyzers- respiratory problems detection Camera- measurements of body temperature, lameness, and injury RFID system and DL model-monitoring feeding and drinking behavior Microfluidics- metabolic disease detection (ketosis) ZigBee-based network- detection of *Salmonella enteritidis* and *Escherichia coli* infections Routine health examination Establishment of diagnostic laboratories Disease-resistance pig breeds Training of farmers

Farm size correlates closely with environmental impact, with some studies indicating that larger farms can mitigate pollution [180–183], while others highlight their negative effects [184,185]. Advanced technologies for manure treatment can produce value-added products, such as biosolids and recycled water [186], while reducing atmospheric emissions [187]. Research suggests that techniques like slurry separation [188–190] and anaerobic digestion [191] are effective in manure management. Sustainable methods include on-farm separation of liquid and solid fractions [192], focusing on reducing N_2_ and P emissions. Integrated livestock-crop systems can enhance environmental sustainability and farm viability by utilizing animal waste as fertilizer, thereby improving soil health [193]. In China, anaerobic digestion systems process about 7% of manure, while the majority is applied directly to agricultural land [191]. However, over-application can exacerbate emissions [194]. Efforts are underway to produce bioenergy and organic materials from animal waste, leveraging methods like gasification and pyrolysis for sustainable waste management (Fig. 6) [195,196].

PLF tools enable real-time monitoring of individual animals, facilitating health and welfare assessments [85]. While these tools enhance operational efficiency, they may also alter the human-animal relationship by treating animals more as data sources than sentient beings [115]. It is crucial to balance technological application with animal welfare, ensuring that the intrinsic needs of animals are respected. To promote sustainable development in large-scale precision pig farming, it is essential to integrate humane technology designs, provide farmer training, and develop regulatory frameworks. Engaging stakeholders and supporting innovative research will help address ethical concerns associated with PLF tools, ensuring that animals are treated with care while enhancing productivity and environmental sustainability. By recognizing pigs as sentient beings and addressing their behavioral needs, the future of large-scale pig farming can be aligned with sustainable practices that benefit both animal welfare and economic viability. Challenges and proposed methods/technologies for improving pig farming in China are presented in Table 2.

## Conclusion

China is the top nation in both pork production and consumption, with pigs serving as the primary source of meat and sustaining farmers’ livelihoods. Large-scale pig farming is increasingly meeting the nation’s pork demand. Issues such as environmental pollution, lack of available land, sudden outbreaks of pandemic diseases, and heavy reliance on foreign resources for raw feed materials have reduced productivity. PLF technologies use sensors and devices to assist in managing feed, disease surveillance, animal behavior, and welfare monitoring, farm environment control, and manure management. Automation in manure management, coupled with the implementation of wastewater treatment technology, can reduce environmental pollution while simultaneously harnessing the potential of manure as a resource for bioenergy production, thereby paving the way for its utilization in future animal farm operations. Innovation in sustainable energy use by incorporating alternative renewable energy sources, such as photovoltaics and wind, not only reduces production costs but also effectively curtails environmental pollution. Thus, it helps attain sustainable development from both economic and environmental standpoints. Future development of Chinese pig production requires ensuring the steady growth of large-scale precision pig farming.

## List of abbreviations

CO_2_, carbon dioxide; KG, kilogram; N₂, nitrogen; P, phosphorus; RMB, the official currency of China; - %, percent; $, United States Dollar; >, greater than.
